# Potentially Probiotic Lactic Acid Bacteria Strains into Italian-Style Salami: Effects on Ripening and Storage Parameters

**DOI:** 10.3390/foods15173159

**Published:** 2026-09-07

**Authors:** Maria Eduarda Costa Campos, Beatriz Barbosa Cordeiro de Campos, Isabella Maciel Costa, Vinicius Tadeu da Veiga Correia, Cosme Damião Barbosa, José Erick Galindo Gomes, Lara Beatriz Oliveira Mateus, Ana Carolina Nascimento Cidrini Golo, Taynan Jonatha Neves Costa, Bruna Luiza Ferreira Moraes, Washington Azevedo da Silva, Christiano Vieira Pires, Gabriel Augusto Marques Rossi, Cléia Batista Dias, Bruna Maria Salotti Souza

**Affiliations:** 1 Department of Technology and Inspection of Products of Animal Origin, School of Veterinary Medicine, Federal University of Minas Gerais, Belo Horizonte 31270-901, MG, Brazilcleiaornellas@gmail.com (C.B.D.); 2 Agricultural Research Company of Minas Gerais (EPAMIG), São João del-Rei 36301-360, MG, Brazil; 3 Department of Bromatological Analysis, Faculty of Pharmacy, Federal University of Bahia, Salvador 40110-909, BA, Brazil; 4 Laboratory of Microbiology, Enzymatic Technology and Bioproducts, Federal University of Agreste of Pernambuco, Garanhuns 55292-278, PE, Brazil; 5 Department of Food, Faculty of Pharmacy, Federal University of Minas Gerais, Belo Horizonte 31270-901, MG, Brazil; 6 Department of Food Engineering, Federal University of Sao Joao del Rei, Sete Lagoas 35702-031, MG, Brazil; 7 Department of Veterinary Medicine, Vila Velha University, Vila Velha 29102-920, ES, Brazil; gabriel.rossi@uvv.br

**Keywords:** fermentation, functional foods, probiotic, sausage

## Abstract

This study evaluated the effects of incorporating potential probiotic strains into Italian-style salami. Three lactic acid bacteria (LAB) strains were tested, including the commercial strain *Pediococcus pentosaceus* co-cultured with *Staphylococcus xylosus* (C), as well as the LAB strains *Lacticaseibacillus paracasei* (GV17), *Lactococcus lactis* (GV103), and *Pediococcus pentosaceus* (PP). Preliminary tests were conducted to assess the behavior of these strains under different pH values, NaCl concentrations, and preservative conditions. C, GV17, and GV103 exhibited greater viability at pH 5.0 and 5.5, while a 2% NaCl concentration promoted higher LAB growth rates. The addition of preservatives did not inhibit LAB growth. Weight loss during ripening was gradual and became significant from day 12 onward. GV17 and PP exhibited water activity values above 0.90, whereas all four formulations reached moisture contents below 35% and LAB counts above 8.00 log CFU/g after 25 days of ripening. After 45 days of storage, the LAB count in the GV17 formulation was 6.27 log CFU/g. PP exhibited the lightest internal color among the salami formulations, whereas red intensity was similar across all formulations. The use of autochthonous LAB strains significantly reduced hardness and chewiness. Overall, the new LAB strains demonstrated potential for the development of functional meat products, expanding opportunities for innovation in the food sector.

## 1. Introduction

Lactic acid bacteria (LAB) are predominant in fermented foods and have long been used as starter cultures in fermented meat products [1]. The use of probiotic LAB in fermented meat processing has increased due to the health benefits associated with the consumption of these microorganisms [2]. The incorporation of probiotic strains may improve well-being and promote intestinal health in both humans and animals [3,4,5]. Functional foods, including probiotic foods, are defined as foods that provide health benefits beyond their basic nutritional value [6]. These foods have attracted considerable consumer interest, encouraging the food industry to explore their potential to promote health and overall well-being [7]. The LAB most commonly used in these products belong to the genera *Lactobacillus*, *Pediococcus*, *Lactococcus*, and *Enterococcus*; however, *Staphylococcus carnosus* and *Staphylococcus xylosus* are also frequently used by the food industry [8].

Autochthonous LAB strains isolated from animal-derived products are often characterized as potential probiotics. Bis-Souza et al. [2] incorporated potentially probiotic autochthonous LAB strains into buffalo mozzarella and evaluated their use in Italian-style salami production. The authors concluded that products containing *Lacticaseibacillus casei* SJRP66 and SJRP169 exhibited favorable technological characteristics throughout the salami production process. The strains *Lacticaseibacillus paracasei* GV17, *Lactococcus lactis* GV103, and *Pediococcus pentosaceus* PP were previously isolated from Minas artisanal cheese, and their probiotic potential has been demonstrated in previous studies. Antimicrobial susceptibility testing showed that GV17 and GV103 were susceptible to clindamycin, chloramphenicol, and erythromycin, whereas PP was resistant to sulfamethoxazole/trimethoprim and vancomycin. Furthermore, the three strains exhibited hydrophobicity values of 32% and auto-aggregation rates greater than 41% at 4, 37, and 42 °C. All three LAB strains also tolerated simulated gastrointestinal tract conditions, maintaining counts above 8.00 log CFU/mL after the enteric phase [9,10].

Through metabolic processes such as glycolysis, lipolysis, and proteolysis, probiotic bacteria interact with food components, hydrolyzing substrates and releasing organic acids, amino acids, peptides, fatty acids, and bacteriocins [11]. These probiotic-derived bioactive compounds can help reduce the risk of foodborne illness, extend shelf life, control cross-contamination, reduce the need for preservatives, and potentially help preserve the nutritional value of food products [12]. Given the growing interest in incorporating probiotics into meat products, this study aimed to evaluate the effects of adding potentially probiotic strains to Italian-style salami. In addition, the study assessed LAB counts throughout the ripening and storage periods and evaluated the physical and technological characteristics of salami produced with these potentially probiotic strains.

## 2. Materials and Methods

### 2.1. Lactic Acid Bacteria Strains

Three LAB strains were obtained from the Culture Collection of the Department of Technology and Inspection of Animal Products at the Federal University of Minas Gerais (DTIPOA/UFMG, Belo Horizonte, MG, Brazil): *Lacticaseibacillus paracasei* GV17, *Lactococcus lactis* GV103, and *Pediococcus pentosaceus* PP. These strains had previously been isolated from the production environments of raw milk cheeses in different regions: GV17 and GV103 from Minas artisanal cheese produced in the Campo das Vertentes region and PP from Minas artisanal cheese produced in the Serra Geral region, Minas Gerais, Brazil. The probiotic potential of strains GV17 and GV103 [9] and PP [10] had previously been evaluated in vitro.

The LAB strains were cultured in Man–Rogosa–Sharpe (MRS) broth (Biolog, Hayward, CA, USA) and stored at −80 °C in the presence of 20% (*v*/*v*) glycerol. For strain activation, 2% (*v*/*v*) of the thawed LAB culture was inoculated into 10 mL of MRS broth and incubated aerobically at 37 °C for 48 h [13].

### 2.2. In Vitro Growth Kinetics Evaluation Under Fermented Sausage Process Conditions

The growth capacity of the three LAB strains under conditions representative of salami production was evaluated at different pH values (4.5, 5.0, and 5.5), NaCl concentrations (2.0%, 3.75%, and 5.0%), and in the presence of curing salt (a mixture of 90% NaCl, 6% sodium nitrite, and 4% sodium nitrate) at 150 ppm, as well as sodium nitrite at 150 ppm. Traditionally, salami production involves a reduction in pH, which may reach values close to 4.4, exposure to a matrix containing approximately 2–5% sodium chloride, and the addition of sodium nitrite and nitrate, which are used as curing and preservative agents at concentrations established by current legislation [14]. The experimental conditions were designed to individually simulate the main intrinsic factors characteristic of fermented meat products, allowing the tolerance of the selected LAB strains to each factor to be assessed.

The test was conducted in vitro using MRS broth according to the method described by Bis-Souza et al. [15]. Briefly, 20 μL of each LAB culture was inoculated into 180 μL of modified MRS broth in 96-well flat-bottom microtiter plates. The plates were incubated at 15 °C and 25 °C, and optical density at 600 nm (OD600) was measured using a microplate reader (BioTek Instruments^®^, Winooski, VT, USA). OD600 was recorded at 60-min intervals for 6 h and again after 24 h of incubation. All experiments were performed in triplicate (n = 9).

### 2.3. Production of Italian-Style Salami

The LAB strains were activated and harvested by centrifugation at 6000× *g* for 10 min at 4 °C, followed by two washes with sterile saline solution. Each culture was then transferred to a screw-cap tube, and the cell concentration was adjusted to 4.0 on the McFarland scale, corresponding to approximately 9.0 log CFU/g, using a McFarland standard (MS Tecnopon^®^, Piracicaba, Brazil).

The meat used for the production of Italian-style salami was obtained from an establishment inspected by the Federal Inspection Service. Four formulations were prepared in triplicate across three independent production batches, with each batch produced on a different day to account for day-to-day variability. The formulations were as follows: C (control), containing the commercial starter culture T-SPX (Chr. Hansen^®^, Hørsholm, Denmark), composed of *P. pentosaceus* and *S. xylosus*; GV17, containing 50% of the commercial T-SPX culture and 50% of the GV17 culture; GV103, containing 50% of the commercial T-SPX culture and 50% of the GV103 culture; and PP, containing 50% of the commercial T-SPX culture and 50% of the PP culture.

All formulations were prepared using 85 g/100 g pork shoulder and 11.5 g/100 g pork back fat. The pork shoulder and back fat were ground using an electric meat grinder (Britânia^®^, 350 W, Joinville, Brazil) fitted with a 6-mm grinding plate. The following ingredients were added: salt (2.3 g/100 g), sucrose (0.8 g/100 g), sodium nitrite (0.02 g/100 g), curing salt (0.05 g/100 g), garlic powder (0.105 g/100 g), white pepper (0.1 g/100 g), sodium erythorbate (0.1 g/100 g), and the respective bacterial culture at a total concentration of 0.025 g/100 g. The mixture was homogenized in a stainless-steel mixer (Misturadeira Inox Malta^®^, Caxias do Sul, Brazil) for approximately 5 min.

After addition of the inoculum containing the strain of interest, the mixture was stuffed into natural bovine casings (40-mm diameter) and divided into portions of approximately 140 g each. The salami portions were placed in a biological oxygen demand (BOD) incubator (Fanem, model 347 CD, São Paulo, Brazil) under controlled temperature and relative humidity (RH) conditions as follows: 25 °C and 90% RH for 4 days; 23 °C and 88% RH for 3 days; 20 °C and 85% RH for 4 days; 18 °C and 80% RH for 5 days; and 15 °C and 75% RH until day 25. At the beginning of the ripening process, the salami portions from all formulations had an average pH of 5.9.

### 2.4. Ripening Evaluation

Weight was measured on days 3, 6, 12, 18, and 25 of ripening using a calibrated analytical balance (BEL Engineering, S203H, Monza, Milan, Italy). Water activity (a_w) was measured on days 3 and 25 using a water activity meter (LabTouch, Novasina^®^, Lachen, Schwyz, Switzerland). Moisture content was determined by oven-drying the samples at 105 °C until a constant weight was reached on days 3 and 25 of ripening.

### 2.5. Total LAB Count Throughout Ripening and Throughout Shelf Life

LAB populations were determined on days 3, 6, 12, 18, and 25 of ripening. Briefly, 10 g of each sample was aseptically transferred to a sterile container and homogenized with 90 mL of 0.85% sterile saline solution by shaking for 1 min. Serial tenfold dilutions were prepared, and appropriate dilutions were plated in triplicate on MRS agar. The plates were incubated aerobically at 37 °C for 48 h. Bacterial counts were expressed as log CFU/g.

LAB populations were also determined on storage days 1, 15, 30, and 45. The salami samples were stored under dry conditions at room temperature (approximately 26 °C). LAB were enumerated on MRS agar after incubation at 37 °C under aerobic conditions for 48 h, and the results were expressed as log CFU/g. Before storage, the samples were vacuum packaged using a vacuum sealer equipped with a suction nozzle (Baiao Group, model B410, Ubá, MG, Brazil).

### 2.6. Technological Characterization: Color and Texture Evaluation

Instrumental color evaluation was performed at the end of the ripening period using nine replicates for both the internal and external surfaces of the salami. The color parameters (L*, a*, and b*) were measured using a colorimeter (CM-2300d, Konica Minolta, Chiyoda, Japan). Color measurements were performed according to the CIELAB system established by the International Commission on Illumination (CIE), using illuminant D65, a 10° standard observer, an 8-mm aperture, and an 11-mm measurement area.

Instrumental texture analysis was performed at the end of the ripening period using a texture profile analyzer (TA.XTplus, Stable Micro Systems, Godalming, UK). Cylindrical samples measuring 30 mm in diameter and 20 mm in height were subjected to two consecutive compressions to 50% of their original height. The pre-test and test speeds were set at 2.0 and 1.0 mm/s, respectively, with a 5.2-s interval between compressions. A 50-mm-diameter probe was used, with a trigger force of 0.05 N. The texture profile parameters calculated were hardness, springiness, cohesiveness, gumminess, and chewiness [16].

### 2.7. Statistical Analysis

The data were analyzed using descriptive statistics and subjected to analysis of variance (ANOVA) to identify significant differences between treatments. The experimental design was completely randomized (CRD), with four treatments and three replicates per treatment. Product weight was analyzed using the Student–Newman–Keuls (SNK) test. Color, water activity, moisture, and microbial viability during ripening and shelf life were compared using Tukey’s test. Texture was evaluated using Fisher’s least significant difference (LSD) test.

To assess the significance of all tests, treatment means were compared using the appropriate statistical test based on the sensitivity of the coefficient of variation, with a 95% probability of reflecting reality. Statistical analyses were performed using InfoStat software, version 2020 [17]. Predicted means and standard errors were generated from the models, and the least significant difference (LSD) was calculated based on a 5% critical value, allowing for comparisons between treatment means.

## 3. Results

### 3.1. In Vitro Growth Kinetics of Salami Under Process Conditions

**Figure 1:**
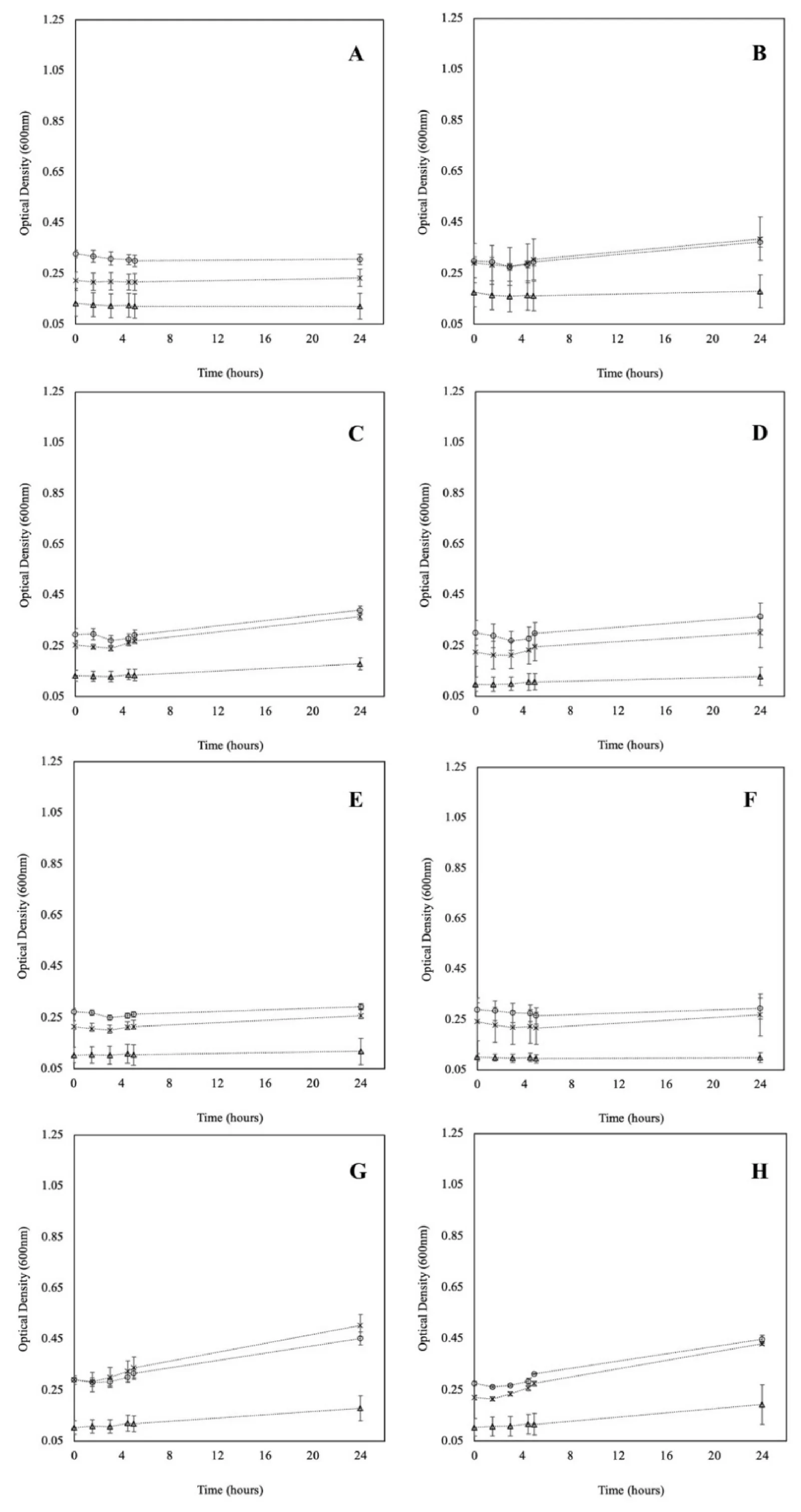
Comparison of the growth of *Lacticaseibacillus paracasei* GV17 (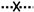), *Lactococcus lactis* GV103 (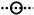) and *Pediococcus pentosaceus* PP (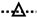) in modified MRS under pH variation 4.5 (**A**), 5.0 (**B**) and 5.5 (**C**), at NaCl concentration 2.0% (**D**), 3.75% (**E**) and 5.0% (**F**) and concentration of sodium nitrite (**G**) and curing salt (**H**), for 24 h at 15 °C.

**Figure 2:**
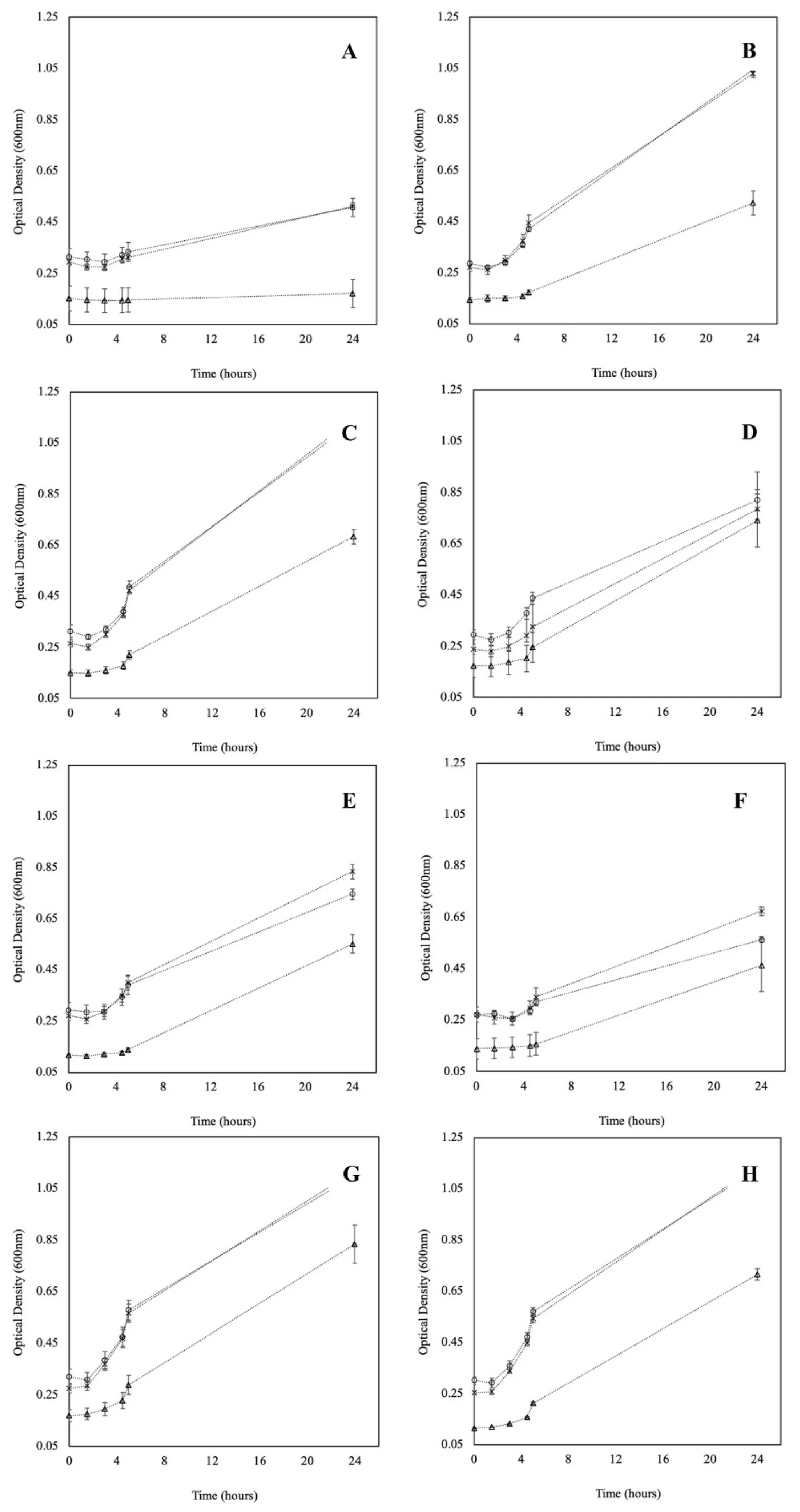
Comparison of the growth of *Lacticaseibacillus paracasei* GV17 (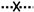), *Lactococcus lactis* GV103 (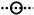) and *Pediococcus pentosaceus* PP (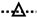) in modified MRS under pH variation 4.5 (**A**), 5.0 (**B**) and 5.5 (**C**), at NaCl concentration 2.0% (**D**), 3.75% (**E**) and 5.0% (**F**) and concentration of sodium nitrite (**G**) and curing salt (**H**), for 24 h at 25 °C.

At 15 °C and pH 4.5, the potentially probiotic strains did not show an increase in growth rate (δ), which was a limiting factor for GV17, GV103, and PP (Figure 1). At 25 °C and pH 4.5, GV17 and GV103 exhibited growth after 24 h (OD_600nm_ = 0.2) (Figure 2). GV17 and GV103 showed higher counts at pH 5.0 and 5.5 at the temperatures tested, with the highest OD_600nm_ value (1.0) observed at 25 °C.

Among the three sodium chloride concentrations tested, 2.0% supported the growth of GV17 and GV103 at both 15 °C and 25 °C, although growth was greater at 25 °C, with OD_600nm_ > 0.6. However, higher sodium chloride concentrations (3.75% and 5%) limited the growth of GV17 and GV103 at 15 °C but not at 25 °C. Concentrations of 150 ppm sodium nitrite and 150 ppm curing salt did not limit growth at the temperatures evaluated, particularly for GV17 and GV103 (Figure 1 and Figure 2), with OD_600nm_ > 0.4. Sodium nitrite is a mandatory additive in the production of Italian-style salami, emphasizing the importance of this evaluation.

### 3.2. Assessment of Ripeness: Weight, Moisture and Water Activity

In the evaluation of the weight of the four different formulations, no differences were observed between treatments (*p* > 0.05) (Table 1). During ripening, significant weight loss (*p* < 0.05) was observed in products from all four treatments. The GV17, GV103, and PP treatments showed significant weight reduction (*p* < 0.05) starting on day 12 of ripening, with weight reduction rates over the 25-day period of 46.74%, 42.86%, and 40.53%, respectively. The control treatment exhibited significant weight loss (*p* < 0.05) starting on day 6 of ripening, with a weight reduction rate of 44.21%.

**Figure 3:**
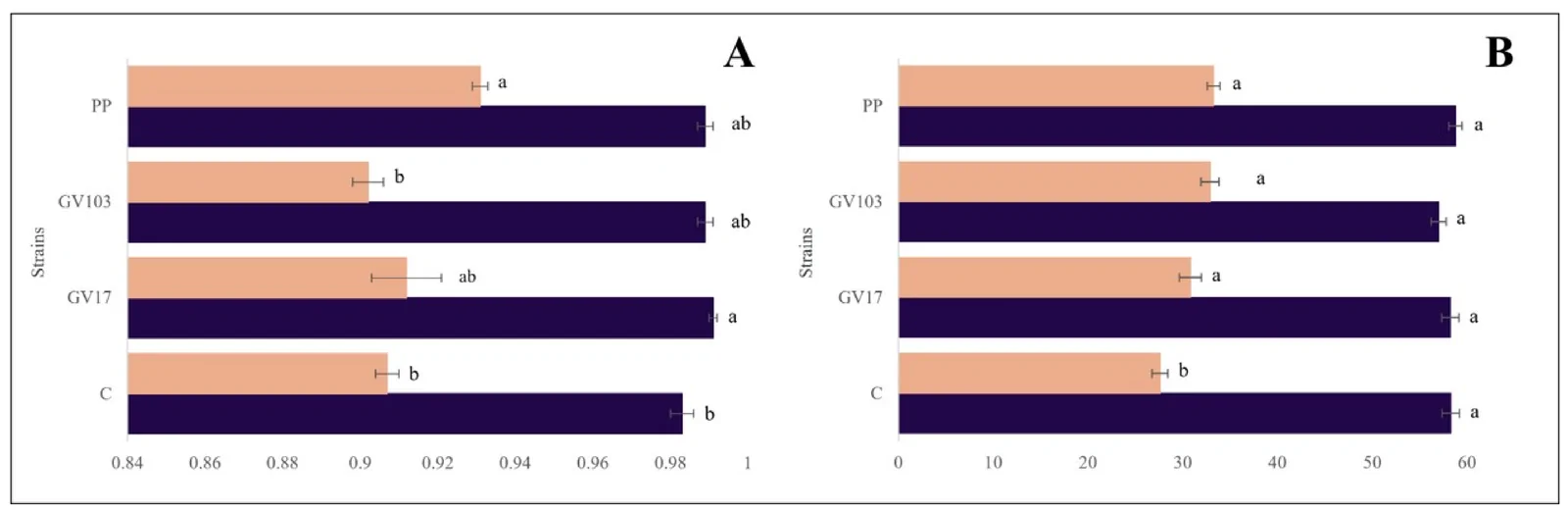
Effect of the addition of lactic acid bacteria, potentially probiotic, on water activity (**A**) and moisture (**B**) of Italian type salami, on the 3rd days (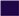) and 25th days (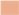) of ripening. Different lowercase letters in the same figure ((**A**) or (**B**)) indicate significant difference between the strains evaluated for the same day, by Tukey’s test (*p* < 0.05). C = *Pediococcus pentosaceus* + *Staphylococcus xylosus*; GV17 = *Lacticaseibacillus paracasei*; GV103 = *Lactococcus lactis*; PP = *Pediococcus pentosaceus*. The results are expressed as mean ± standard error (n = 9).

After 25 days of ripening, GV103 exhibited the lowest A_w_ value (0.902), followed by the control formulation (C = 0.907). GV17 and PP showed A_w_ values greater than 0.910, but with a significant reduction (*p* < 0.05) compared with the first day of maturation (Figure 3A). The PP treatment showed a higher result (*p* < 0.05) compared with the C and GV103 treatments.

### 3.3. Total LAB Count Throughout Ripening

On the 3rd day of ripening, LAB viability was greater than 9.0 log CFU/g in salami from all four treatments (Table 2). GV103 exhibited a count of 11.53 log CFU/g on day 3; however, on day 6 of ripening, it showed the greatest reduction in count, with a decrease of approximately 2.8 log CFU/g.

At the end of the 25-day ripening period, salami from all treatments had LAB counts above 8.0 log CFU/g. Over the 25-day period, there was no significant difference (*p* > 0.05) in LAB counts between formulations C and PP. GV103 showed a significant difference in counts (*p* < 0.05) between day 3 and the other days of ripening. GV17 exhibited the greatest variation throughout the ripening period. The commercial strain (C) exhibited a variation in LAB counts from 8.20 log CFU/g on day 12 to 9.56 log CFU/g on day 6; however, no significant differences were observed among the ripening times.

### 3.4. Technological Characterization of Color and Texture

When evaluating the internal color, the formulation with the addition of PP resulted in a lighter salami compared with the commercial strain (Table 3). The intensity of the red color (a* value) inside the salami was higher in GV103 (*p* < 0.05) than in PP but did not differ (*p* > 0.05) from C and GV17. The intensity of the yellow color (b* value) was similar among the GV17, GV103, and PP formulations.

When evaluating the external color, a difference (*p* < 0.05) in luminosity (L*) was observed between the salami formulations containing GV17 and GV103. The salami containing GV17 exhibited a darker coloration. The intensity of the red color was similar (*p* > 0.05) among all formulations, while the intensity of the yellow color was similar (*p* < 0.05) between the C and GV103 formulations and among the GV17, GV103, and PP formulations (Table 3).

### 3.5. LAB Counts Throughout Shelf Life

**Figure 4:**
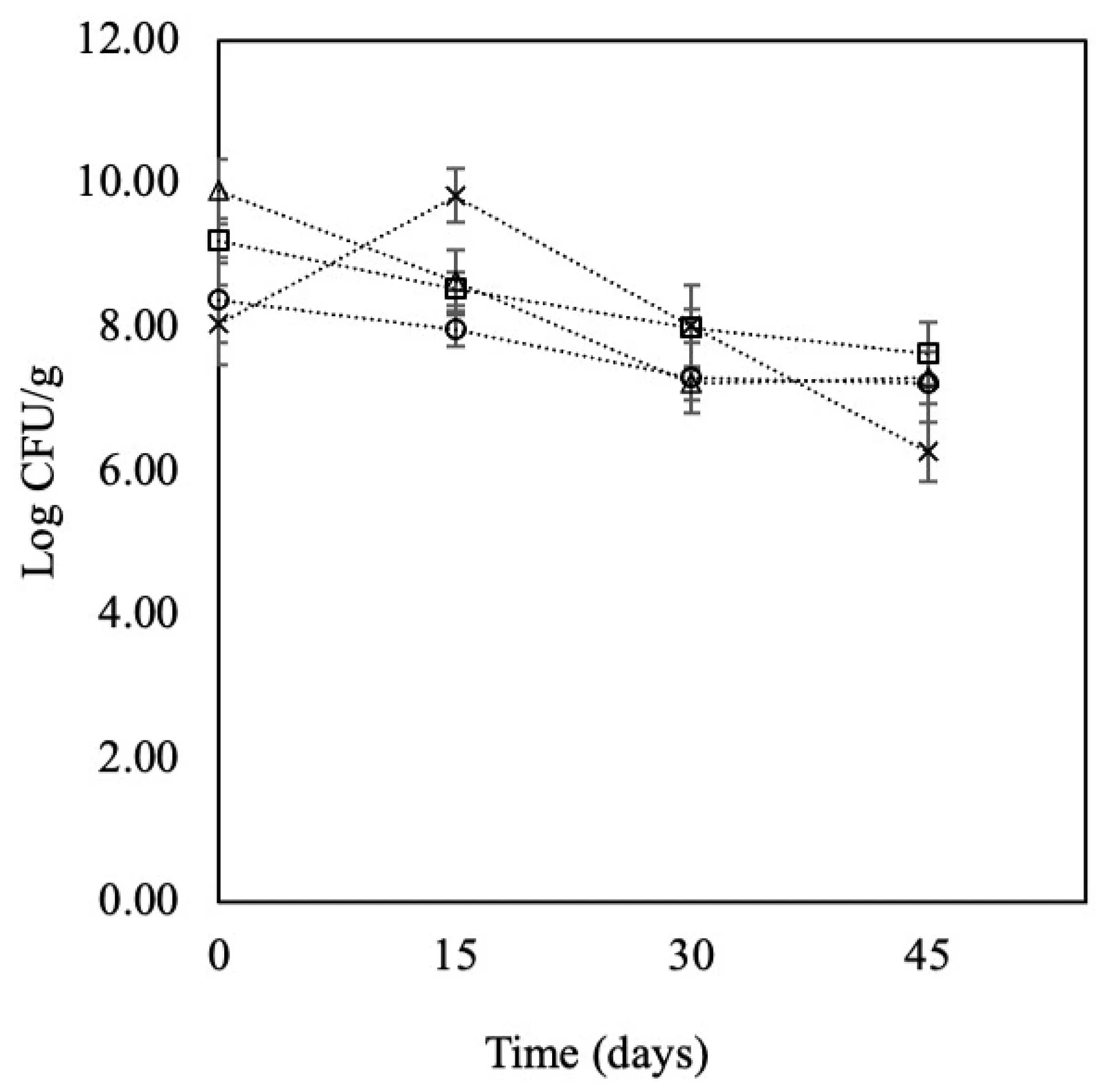
Viability of lactic acid bacteria in the different strains tested, (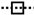) *Pediococcus pentosaceus* + *Staphylococcus xylosus* C; (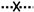) *Lacticaseibacillus paracasei* GV17; (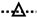) *Lactococcus lactis* GV103; (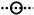) *Pediococcus pentosaceus* PP, throughout the shelf life of Italian-style salami. The *Y*-axis represents the colony-forming units per g and the *X*-axis represents the days of shelf life (Day 0, 15th, 30th and 45th). The results are expressed as mean ± standard error (n = 9).

All tested formulations showed LAB counts greater than 6.0 log CFU/g after 45 days, with the highest value observed in formulation C (7.63 log CFU/g), followed by PP (7.31 log CFU/g) and GV103 (7.23 log CFU/g). Both C and GV103 maintained LAB counts greater than 7.0 log CFU/g throughout the 45-day shelf life of the product, with no significant differences (*p* > 0.05) (Figure 4). The microbial counts observed throughout the shelf life may be attributed to the presence of the commercial culture incorporated into all tested formulations.

The salami containing GV17 showed a reduction of approximately 2.0 log CFU/g from the first day to day 45 of shelf life but remained above 6.0 log CFU/g. The potentially probiotic strain PP showed a reduction of more than 3.0 log CFU/g after 30 days; however, by day 45, it reached a viability of 7.31 log CFU/g. *Pediococcus pentosaceus* is considered a starter LAB commonly used in meat fermentation. Its early involvement contributes to accelerated acidification through the production of lactic and acetic acids [18]. The initial activity of *Pediococcus* spp. may have contributed to the reduction in microbial counts observed at the end of the product’s shelf life.

## 4. Discussion

During the technological processes used in the food industry to produce various fermented products, LAB are exposed to environmental stresses, including acidic, osmotic, thermal, and even oxidative stress [19]. These factors can affect their growth, metabolic activity, and overall efficiency during fermentation [20]. Therefore, understanding how native LAB behave within the salami matrix is critically important.

In all tests conducted to evaluate growth kinetics, whether involving variations in pH, preservative concentration, or sodium chloride concentration, the PP strain, despite showing growth under certain conditions, consistently exhibited a lower growth rate than GV17 and GV103. The tested strains, particularly GV17 and GV103, exhibited better performance under the evaluated conditions when incubated at 25 °C.

Although GV17, GV103, and PP were isolated from Minas artisanal cheese, which has an acidic pH, the LAB strains exhibited a low multiplication rate at pH 4.5, indicating that this condition was a limiting factor. LAB are important microorganisms that produce lactic acid as a fermentation byproduct. They are widely distributed in various environments and are used in the production of fermented plant- and animal-based products [21]. Their adaptation to acidic conditions suggests better adaptation to acidic environments. Previous tests demonstrated that GV17 and GV103 tolerated simulated gastrointestinal tract (GIT) conditions at pH 2.5, maintaining viability greater than 8.0 log CFU/mL after the gastric and intestinal phases [9].

Nitrite is widely used in the food industry to enhance flavor, promote the development and stabilization of the characteristic color of cured meat products, and exert preservative effects, with maximum permitted levels varying among countries. The Food and Drug Administration (FDA) allows the use of sodium nitrite as a preservative in meat products at concentrations not exceeding 200 ppm [22]. According to EU regulations, the maximum allowable nitrite concentration is 150 ppm [23]. In Brazil, the maximum concentration of sodium nitrite in meat products available for sale cannot exceed 150 ppm [24].

When sodium nitrite is used as a preservative in fermented salami, it is crucial that LAB can still proliferate in its presence, making this an important evaluation criterion. LAB can degrade nitrite through three main pathways: (i) reduction by nitrite reductase, (ii) acid degradation at pH > 4.5, and (iii) enzymatic degradation, the latter being the dominant mechanism [25]. The distinctive enzyme system of LAB catalyzes reactions involving glucose to produce acids and enhances the degradation of nitrite reductase. This process reduces biogenic amine levels and inhibits the formation of N-nitrosamines, ultimately bringing nitrite levels down to safe limits [26]. Under acidic conditions, nitrites are converted to nitrous acid, which can react with secondary amines to form N-nitrosamines.

An additional limiting factor for LAB growth was the increased sodium chloride concentration. While NaCl levels between 1.0% and 2.5% are considered low and have been shown to stimulate LAB growth and lactic acid production, higher concentrations can inhibit LAB proliferation [27,28].

Microbial development was similar at both 15 °C and 25 °C; however, higher temperatures can accelerate the acidification process, thereby inhibiting the growth of bacteria from the Micrococcaceae family. These bacteria play a crucial role in reducing nitrate to nitrite, which is essential for the development of sensory characteristics such as color, flavor, and aroma [18].

In the technological evaluation of LAB applied to Italian-style salami, the moisture content after 25 days of ripening in all four treatments was in accordance with Brazilian legislation, which establishes a maximum moisture content of 35% for Italian-style salami [29] (Figure 3B). Regarding moisture content, the control formulation showed a reduction (*p* < 0.05) compared with treatments GV17 (30.81%), GV103 (32.90%), and PP (33.25%), reaching a value of 27.59%.

Weight reduction, water activity, and moisture content in salami are natural processes that occur during maturation and are influenced by various factors, including packaging parameters, formulation, drying process, diameter, and ripening time [30]. Salami weight loss is consistent throughout ripening [31], and this effect was observed in all four formulations tested (Table 1 and Figure 3).

Water activity also decreases gradually during ripening. Espinales et al. [31] evaluated various salami formulations aimed at improving the nutritional, functional, and technological quality of the product. They observed that the initial water activity ranged from 0.985 to 0.988, with a reduction to below 0.850 after 25 days of maturation.

In response to reduced moisture and A_w_, LAB may develop self-regulatory mechanisms to mitigate the effects of these adverse conditions and ensure survival [32]. The stress response in LAB depends on the type of stress encountered and the specific bacterial species involved, allowing their multiplication to continue. LAB can activate adaptive mechanisms to mitigate the effects of adverse conditions to which they are exposed, thereby enhancing their survival [20]. These stress responses are often mediated by changes in gene expression, which can affect cell division, transport processes, DNA metabolism, and even membrane composition [33].

During the initial phase of ripening of Italian-style salami, the availability of fermentable sugars is higher, temperature conditions are favorable for LAB multiplication, and microbial competition is minimal [34]. When LAB are added as starter cultures, they can multiply rapidly and dominate the microbiota. As the ripening process progresses, a reduction in A_w_, which ranged from 0.89 (GV103) to 0.93 (PP) (Figure 3A), along with nutrient depletion, may limit bacterial replication, potentially leading to a decline in LAB counts. The reduction observed in the GV17 line on day 18 of maturation may be attributed to these factors.

Significant differences were observed among the tested strains. On days 6 and 25, no significant differences (*p* > 0.05) were found among the four formulations. GV17 and PP showed significant similarity (*p* < 0.05) to the control formulation (C). The PP formulation exhibited lower weight loss, a smaller reduction in water activity and moisture content, and a higher viable cell count after 25 days of maturation. The variation in weight among salami pieces, as observed in the PP formulation on days 18 and 25, may be attributed to differences in moisture loss among the pieces [35].

Color development is influenced by several reactions that occur during salami maturation [36]. Brightness is associated with a decrease in the amount of reflected light, which is due to moisture loss [37]. Therefore, a darker salami may indicate less moisture loss. The PP formulation showed a higher L* value compared with C (Table 3). Higher L* values indicate greater surface luminosity of the salami and may be associated with changes in the meat matrix during fermentation, particularly acidification and modifications in protein structure and water distribution, which can increase light scattering [38].

The red color (a*) is associated with the reduction of nitrite to nitric oxide and the formation of nitrosomyoglobin, which is responsible for the desired pigmentation in cured meat products [39]. Thus, the greater intensity of the internal red color in the GV103 formulation may be linked to enhanced oxidation of the product or to the fact that some strains of *Lactococcus lactis* are known to produce zinc protoporphyrin IX (*ZnPP*), which may contribute to the bright red color of cured meat products [40].

The addition of potentially probiotic strains to Italian-style salami resulted in a significant decrease (*p* < 0.05) in hardness and chewiness compared with formulation C (Table 4). No significant differences (*p* > 0.05) were observed among the formulations in terms of elasticity, cohesiveness, and resilience. Salami produced with the addition of GV17 and C showed similar gumminess (*p* < 0.05).

Texture is a key quality parameter for food products. The reduction in hardness and chewiness in formulations containing probiotic strains suggests that the addition of these strains altered the structural matrix of the salami, making it less firm (or softer) and consequently easier to chew. These changes are likely related to the protein composition or may be attributed to the action of the strains in hydrolyzing specific components, such as proteins or fats, during the fermentation process. The lack of variation in elasticity, cohesiveness, and resilience suggests that the strains do not compromise the integrity of the product. In addition to the functional benefits, the incorporation of probiotic strains may positively impact palatability if consumer preference favors a softer texture and improved chewiness.

The differences in hardness observed among treatments may be attributed to the combined effects of acidification, moisture loss, protein aggregation, and proteolysis during fermentation and maturation. LAB strains can influence these processes through their distinct acidifying and proteolytic activities, thereby affecting the development of the protein matrix and the final texture of the fermented meat product [41]. Gomes et al. [42] applied the microencapsulated probiotic *Bifidobacterium animalis* BB-12 in the production of Italian salami and found no significant differences in dryness, elasticity, cohesiveness, or chewiness between the control formulation without the probiotic and the formulations containing the probiotic strain.

The final factor evaluated in the salami was the viability of LAB throughout the 45-day shelf life. The widely accepted definition of probiotic microorganisms refers to those that, when administered in adequate amounts, provide health benefits to the host [43,44].

Thus, LAB strains characterized as potentially probiotic must remain viable in the food until the end of its shelf life and subsequently withstand adverse GIT conditions. Previous studies have demonstrated that GV17, GV103 [9], and PP [10] resisted simulated GIT conditions and, after 45 days of storage under vacuum packaging, maintained viability greater than 6.0 log CFU/mL.

Cell loss may occur during processing, transportation, and even storage. A viable probiotic intake of 6.00 to 9.00 log CFU/mL or g is considered the minimum therapeutic level that can confer health benefits to humans [45]. Therefore, the developed formulations meet the required therapeutic level after 45 days of storage.

The higher microbial count observed in the control formulation (C) (7.77 log CFU/g) at the end of the 45-day shelf life may be attributed to the better adaptation of the strains to the food matrix. Commercial LAB strains generally exhibit greater multiplication due to their enhanced ability to adapt to the food environment [46].

Bis-Souza et al. [15] applied potentially probiotic *Lactobacillus* strains to low-fat salami formulations supplemented with fructooligosaccharides (FOSs) and observed LAB viability greater than 8.0 log CFU/g after 18 days of ripening. Additionally, the authors reported a higher L* value (*p* < 0.05) in the control formulation compared with the formulations with reduced fat, added FOS, and autochthonous LAB from buffalo mozzarella.

LAB counts in formulations C and PP remained higher than those observed in GV17 and GV103 throughout ripening, and this difference persisted throughout the entire shelf life of the salami (Table 2 and Figure 4). This may be attributed to the greater adaptation of commercial strains to the ripening and storage conditions of fermented meat products [15]. Furthermore, *P. pentosaceus* is considered well adapted to the processing conditions of fermented meat products [47]. Among 174 LAB isolates evaluated, Nanasombat et al. [48] reported that *P. pentosaceus* P0805 showed the best adaptation to the fermentation conditions of meat products. A relevant aspect for future research involves investigating the impact of potentially probiotic strains on the sensory attributes of fermented meat products, including flavor, aroma, texture, and appearance. A comprehensive understanding of these attributes would provide valuable insights into the contributions of these strains to overall product quality and their potential application in functional foods.

## 5. Conclusions

The incorporation of potentially probiotic lactic acid bacteria, including *Lacticaseibacillus paracasei* GV17, *Lactococcus lactis* GV103, and *Pediococcus pentosaceus* PP, was effective in the production of Italian-style salami when combined with commercial starter cultures. LAB counts remained at favorable levels throughout the product’s shelf life, indicating the ability of these strains to persist in the fermented meat matrix.

Based on the parameters evaluated, the combination of autochthonous, potentially probiotic LAB with commercial starter cultures resulted in favorable technological characteristics. Overall, the incorporation of these potentially probiotic strains represents a promising approach for the development of Italian-style salami with potential functional properties while maintaining desirable technological characteristics. Further studies involving comprehensive physicochemical, compositional, and sensory analyses are warranted to fully characterize product quality and assess consumer acceptance.

## Figures and Tables

**Table 1:** Effect of adding potentially probiotic lactic acid bacteria on the weight (g) of Italian-type salami during the ripening process.

Strains		3rd	6th	12th	18th	25th
C	*P. pentosaceus* + *S. xylosus*	139.01 ^a^ ± 6.3	119.72 ^b^ ± 5.3	95.59 ^c^ ± 5.4	79.25 ^c^ ± 5.2	77.55 ^c^ ± 5.1
GV17	*Lbs. paracasei*	156.15 ^a^ ± 9.4	140.00 ^a^ ± 9.1	107.57 ^b^ ± 4.2	86.12 ^c^ ± 2.3	83.15 ^c^ ± 2.2
GV103	*L. lactis*	145.01 ^a^ ± 5.8	134.87 ^a^ ± 4.8	103.06 ^b^ ± 3.0	85.18 ^c^ ± 2.5	82.85 ^c^ ± 2.3
PP	*P. pentosaceus*	134.38 ^a^ ± 8.2	121.85 ^a^ ± 7.6	97.09 ^b^ ± 5.9	73.98 ^b^ ± 9.9	79.91 ^b^ ± 5.4

Different lowercase letters in the line indicate significant difference between the times evaluated for the same LAB strain, by the SNK test (*p* < 0.05). The results are expressed as mean ± standard error (n = 6).

**Table 2:** Viability of lactic acid bacteria, potentially probiotic, during the ripening period of Italian-style salami (log CFU/g).

Strains		3rd	6th	12th	18th	25th
C	*P. pentosaceus* + *S. xylosus*	9.41 ^Ab^ ± 0.37	9.56 ^Aa^ ± 0.45	8.20 ^Ab^ ± 0.37	8.82 ^Aab^ ± 0.48	9.20 ^Aa^ ± 0.31
GV17	*Lbs. paracasei*	10.22 ^Ab^ ± 0.26	9.28 ^ABa^ ± 0.32	9.60 ^Aa^ ± 0.36	7.22 ^Cb^ ± 0.23	8.04 ^BCa^ ± 0.55
GV103	*L. lactis*	11.53 ^Aa^ ± 0.18	8.68 ^Ba^ ± 0.60	9.88 ^ABa^ ± 0.18	9.14 ^Ba^ ± 0.49	8.38 ^Ba^ ± 0.59
PP	*P. pentosaceus*	9.54 ^Ab^ ± 0.46	9.09 ^Aa^ ± 0.59	10.03 ^Aa^ ± 0.16	8.80 ^Aab^ ± 0.48	9.90 ^Aa^ ± 0.45

Different lowercase letters in the column indicate significant difference between strains and different uppercase letters in the row indicate significant difference between the times evaluated for the same LAB strain, by Tukey’s test (*p* < 0.05). The results are expressed as mean ± standard error (n = 9).

**Table 3:** Effect of the addition of lactic acid bacteria, potentially probiotic, on the internal and external color parameters of Italian-type salami.

Strains		Internal Coloring			External Coloring		
		L*	a*	b*	L*	a*	b*
C	*P. pentosaceus* + *S. xylosus*	35.5 ^b^ ± 2.1	8.5 ^ab^ ± 1.1	23.8 ^a^ ± 0.9	29.8 ^ab^ ± 1.1	10.1 ^a^ ± 0.7	28.7 ^a^ ± 1.0
GV17	*Lbs. paracasei*	41.8 ^ab^ ± 1.7	7.5 ^ab^ ± 1.1	21.3 ^ab^ ± 0.8	27.4 ^b^ ± 1.2	10.1 ^a^ ± 0.6	25.1 ^b^ ± 1.1
GV103	*L. lactis*	40.0 ^ab^ ± 1.6	10.6 ^a^ ± 0.7	19.7 ^b^ ± 0.9	34.5 ^a^ ± 1.5	9.8 ^a^ ± 0.7	25.7 ^ab^ ± 0.8
PP	*P. pentosaceus*	42.5 ^a^ ± 2.0	6.6 ^b^ ± 0.8	23.6 ^a^ ± 0.9	29.5 ^ab^ ± 1.7	10.1 ^a^ ± 0.6	23.9 ^b^ ± 0.6

Different lowercase letters in the column indicate significant difference between strains by Tukey’s test (*p* < 0.05). Results are expressed as mean ± standard error (n = 27).

**Table 4:** Effect of the addition of lactic acid bacteria, potentially probiotic, on the texture parameters of Italian-type salami.

Parameter	C	GV17	GV103	PP
Hardness	73.34 ^a^ ± 20.57	27.10 ^b^ ± 21.78	42.82 ^b^ ± 11.34	37.50 ^b^ ± 4.35
Springiness	0.30 ^a^ ± 0.02	0.33 ^a^ ± 0.01	0.32 ^a^ ± 0.01	0.32 ^a^ ± 0.02
Cohesiveness	0.27 ^a^ ± 0.04	0.29 ^a^ ± 0.01	0.29 ^a^ ± 0.02	0.29 ^a^ ± 0.02
Gumminess	23.50 ^ab^ ± 3.27	27.20 ^a^ ± 5.91	14.82 ^bc^ ± 2.61	10.90 ^c^ ± 1.58
Chewiness	7.59 ^a^ ± 1.26	2.66 ^b^ ± 1.75	3.53 ^b^ ± 0.73	3.64 ^b^ ± 0.74
Resilience	0.08 ^a^ ± 0.01	0.07 ^a^ ± 0.00	0.07 ^a^ ± 0.00	0.07 ^a^ ± 0.00

Different lowercase letters indicate significant difference between LAB strains by Fisher’s LSD test (*p* < 0.05). Results are expressed as mean ± standard error (n = 6). C: *Pediococcus pentosaceus* + *Staphylococcus xylosus*, GV17: *Lacticaseibacillus paracasei,* GV103: *Lactococcus lactis* and PP: *Pediococcus pentosaceus*.

## Data Availability

The datasets generated during and/or analyzed during the current study are available from the corresponding author on reasonable request.
